# Supplementary material for: Questionnaire-Based Study Evaluating the Hand Hygiene Practices and the Impact of Disinfection in the COVID-19 Pandemic on Hand Skin Conditions in Poland
Source: J Clin Med. 2022 Dec 27;12(1):195. doi: 10.3390/jcm12010195 (PMC9821516; doi:10.3390/jcm12010195)
Supplement: Supplementary file 1 [file jcm-12-00195-s001.zip › jcm-2095053-supplementary.pdf]

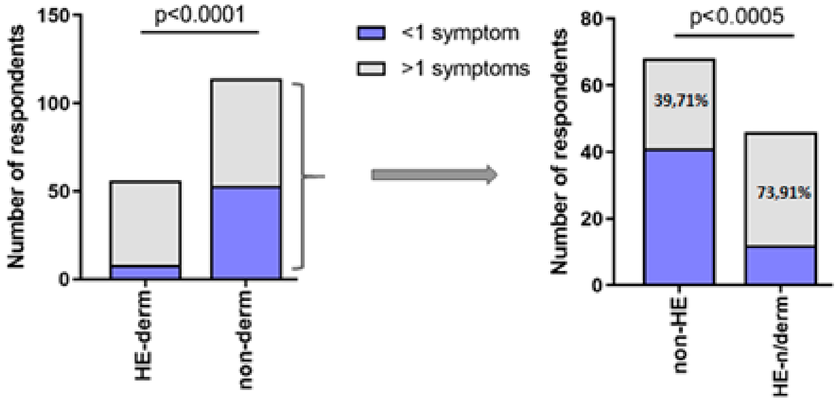

Figure S1. Number of symptoms reported by HE-derm and non-derm groups (distinguishing non-HE, and HE-n/derm groups) immediately after application of the disinfectant.

In the study, **non-derm** group was divided into two subgroups: **non-HE** and **HE-n/derm** group. Respondents from HE-n/derm observed significantly more new symptoms immediately after application of the disinfectant compared to non-HE (*p<0.0005*). Most of respondents from non-HE (nearly 74%) had 1 or less new symptom.

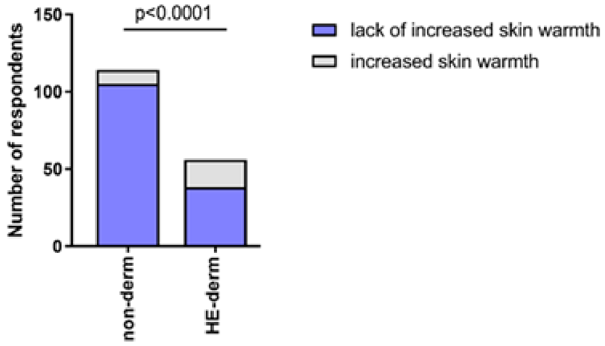

Figure S2. The frequency of increased skin warmth reported by HE-derm group and non-derm group immediately after application of the disinfectant.

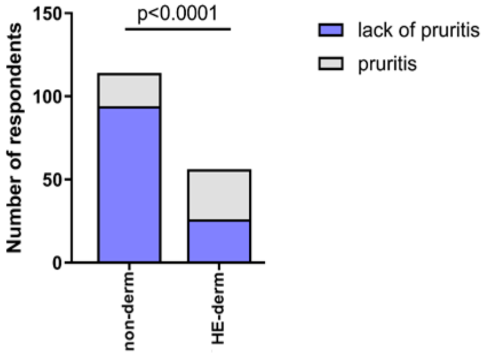

Figure S3. The frequency of pruritis reported by HE-derm group and non-derm group immediately after application of the disinfectant.

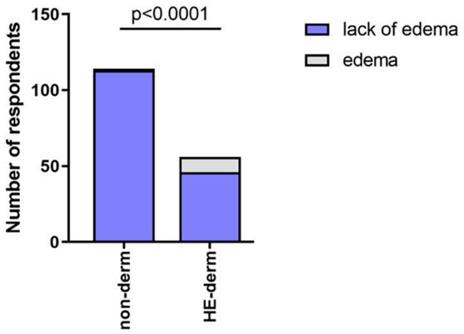

Figure S4. The frequency of edema reported by HE-derm group and non-derm group immediately after application of the disinfectant.

The following symptoms were more frequent in HE-derm group: pruritus (HE-derm group: n=30/56, 53.57%, non-derm group: n=20/114, 17.54%; *p <0.00001*), increased skin warmth (HE-derm group: n=18/56, 32.14%, non-derm group: n=9/114, 7.89%; *p <0.000048*) and edema (HE-derm group: n=10/56, 17.86%, non-derm group: n=1/114, 0.88%; *p <0.000023*). These symptoms disappeared shortly after disinfection.

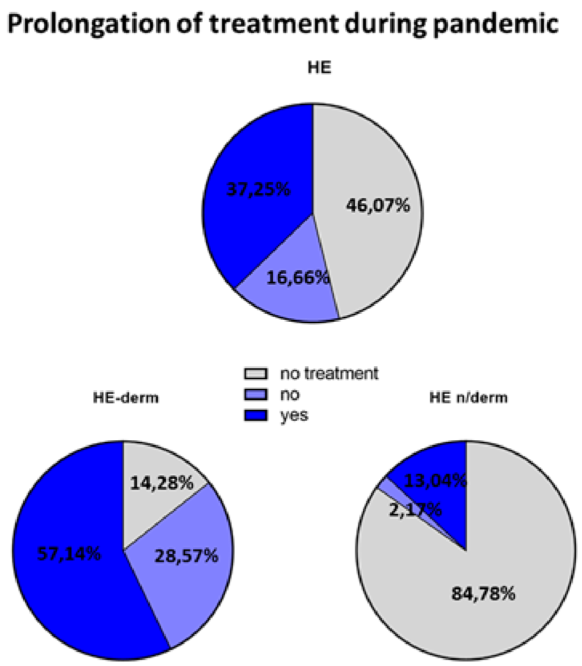

Figure S5. Comparison of prolongation of treatment in the study groups during pandemic.

In HE group nearly 40% of respondents reported prolongation of treatment during pandemic, however a significant part of those persons are from HE-derm group. In HE-derm group, the prolongation was observed by 57.14%, and in HE n/derm, 13.04%, respectively.

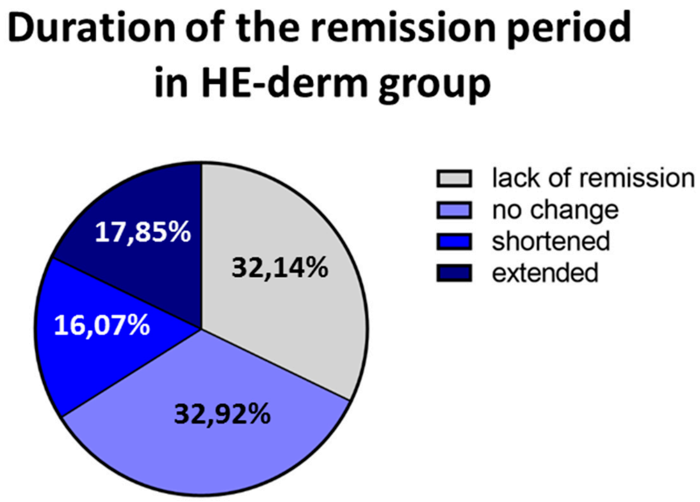

Figure S6. The duration of the remission period in HE-derm group during pandemic.

Nearly 50% subjects from HE-derm group (16.07% shortened, 32.14% lack of remission) observed a deterioration in the course of the hand skin disease during the pandemic. Only 17.85% respondents perceived extended remission period.

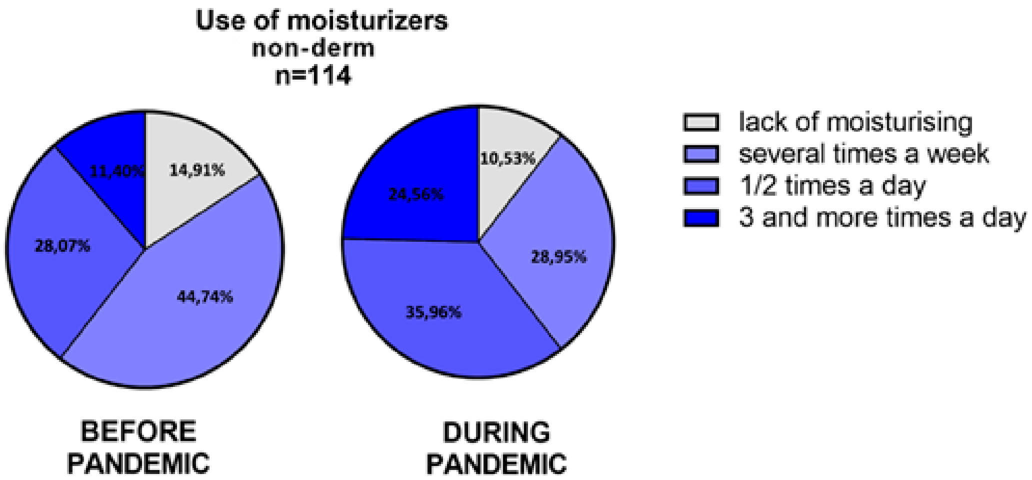

Figure S7. The frequency of moisturizing the skin of the hands in the non-derm group before and during the pandemic.

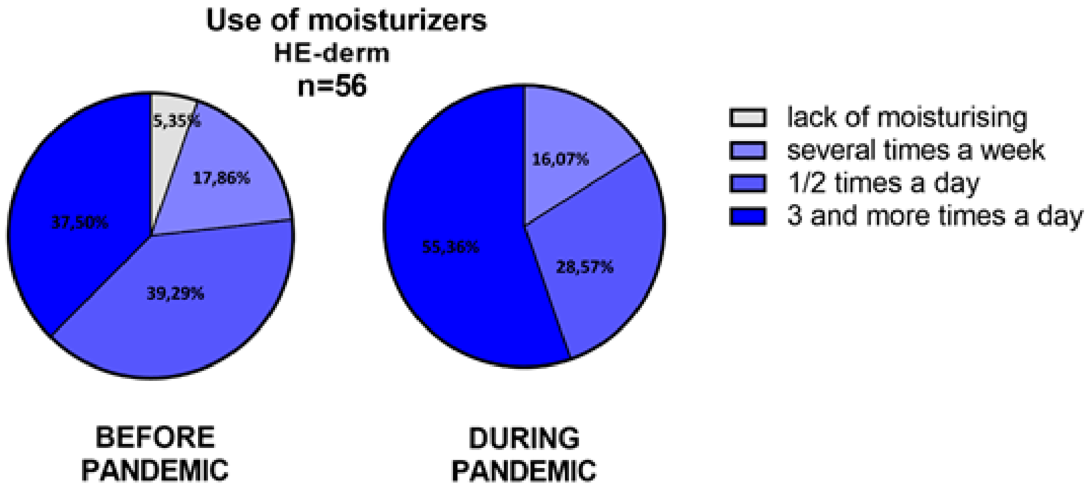

Figure S8. The frequency of moisturizing the skin of the hands in the HE-derm group before and during the pandemic.

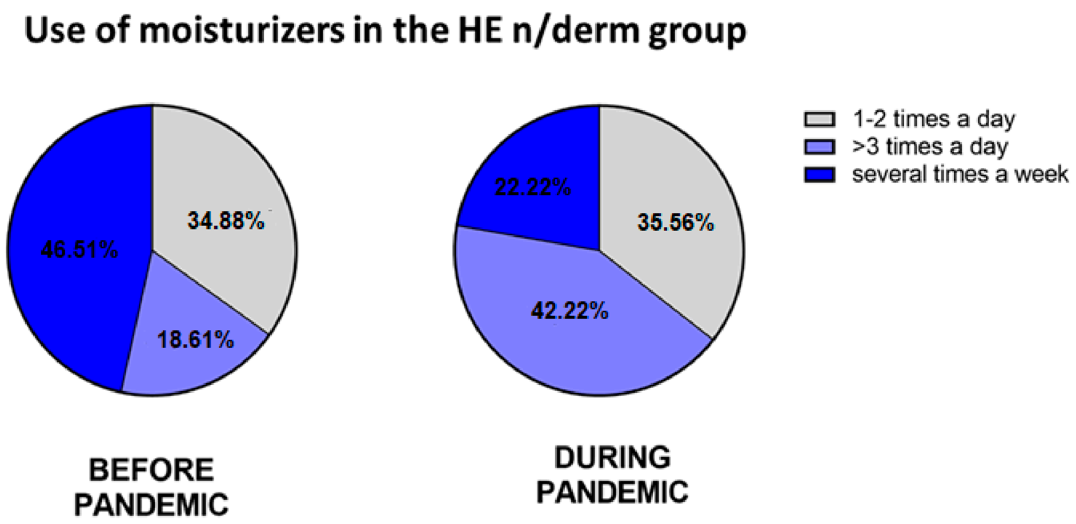

Figure S9. The frequency of moisturizing the skin of the hands in the HE n/derm group before and during the pandemic.

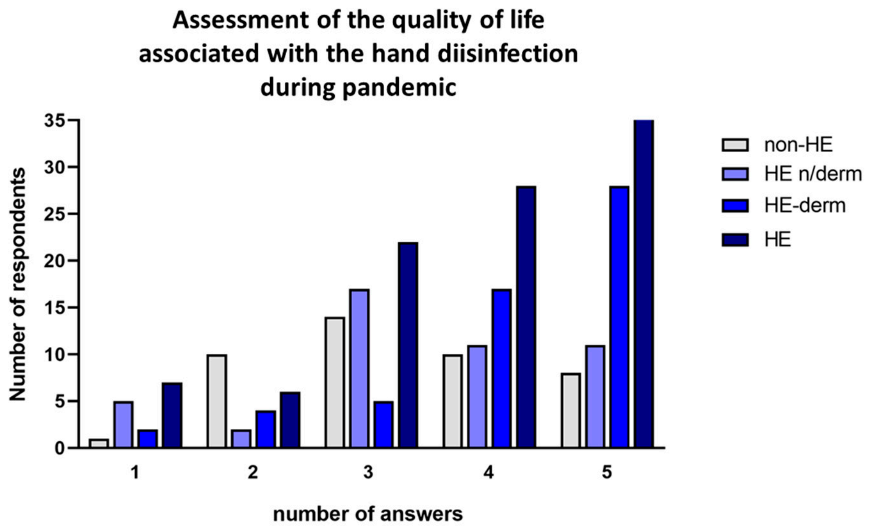

Figure S10. Assessment of the quality of life associated with the hand disinfection in study groups during pandemic. 1- disinfection had no negative effect on the quality of life, 5 – a significant impact of disinfection on the lowering quality of life.

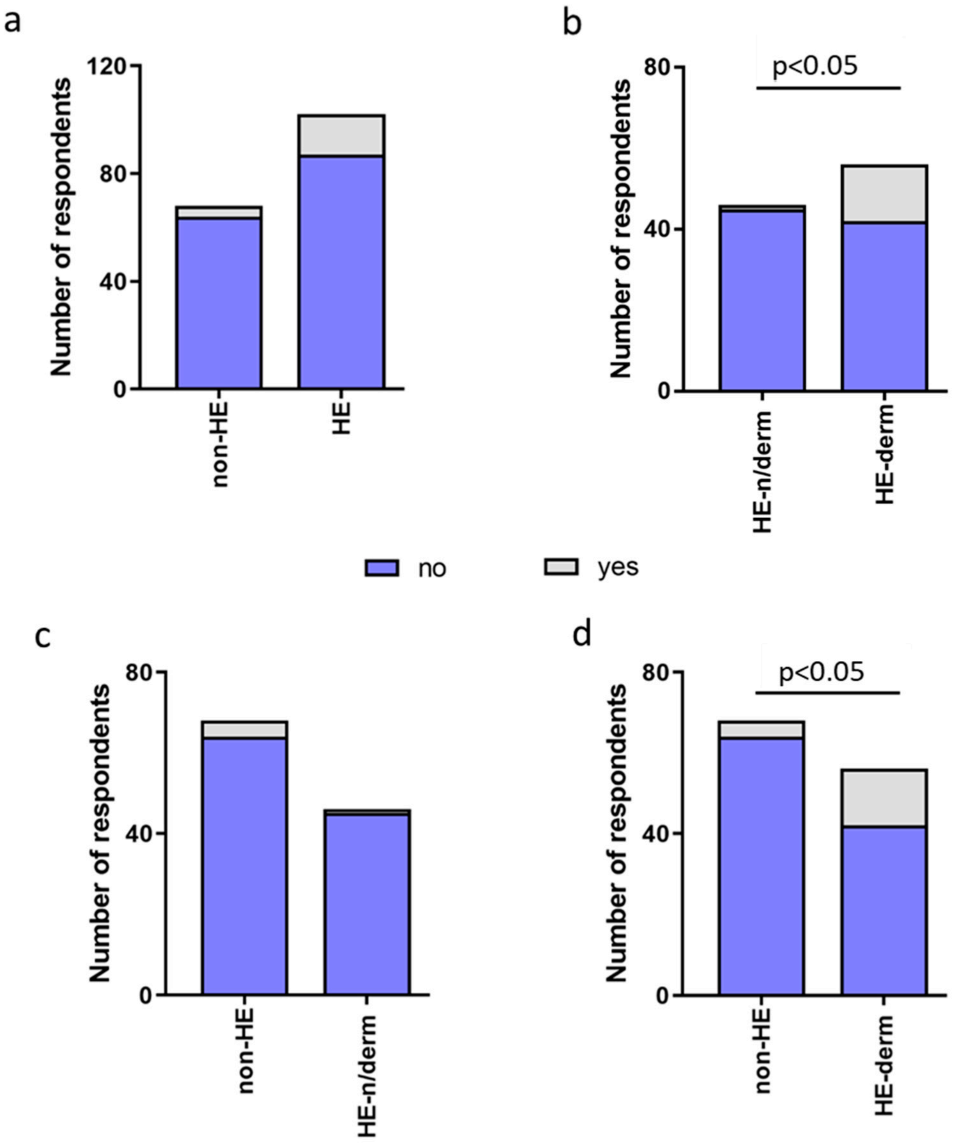

Figure S11. Comparison of the prevalence of skin infections in study groups before the pandemic: (a) non-HE vs HE; (b) HE n/derm vs HE-derm; (c) non-HE vs HE n/derm; (d) non-HE vs HE-derm.

Before the pandemic, significantly more frequent of skin infections in the HE-derm group compared to HE-n/derm and non-HE was observed (*p<0.05*, *p<0.05*, respectively). However, there were no differences between the group non-HE and groups HE and HE-n/derm.

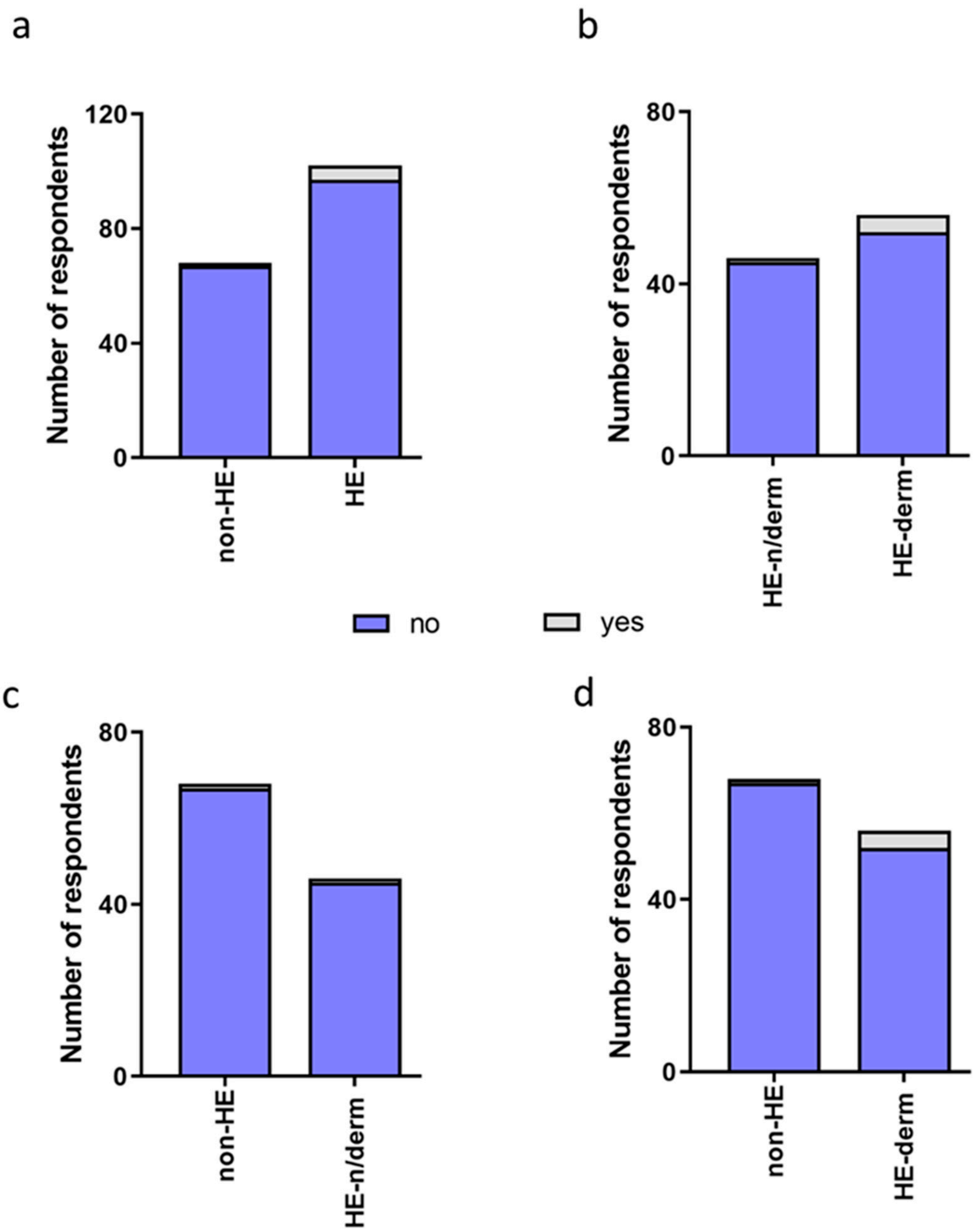

Figure S12. Comparison of the prevalence of skin infections in study groups during the pandemic: (a) non-HE vs HE; (b) HE n/derm vs HE-derm; (c) non-HE vs HE n/derm; (d) non-HE vs HE-derm.
