# Supplementary material for: Questionnaire-Based Study Evaluating the Hand Hygiene Practices and the Impact of Disinfection in the COVID-19 Pandemic on Hand Skin Conditions in Poland
Source: J Clin Med. 2022 Dec 27;12(1):195. doi: 10.3390/jcm12010195 (PMC9821516; doi:10.3390/jcm12010195)
Supplement: Supplementary file 1 [file jcm-12-00195-s001.zip › Table S1.pdf]

**Table S1. –** The questions included in the survey.

| Part of the survey | Survey questions |
|---|---|
| **General questions about demographic characteristics of the study group** | 1. Gender<br>2. Age<br>3. Education and/or profession<br>4. Residence |
| **Questions about dermatological history** | 1. Have you ever suffered from hand skin diseases in the past, and if so, what diagnosis was made?<br>2. Do you currently suffer from hand skin diseases, and if so, what is the diagnosis?<br>3. Have you had patch tests performed or biopsy to confirm hand skin disease? If the results of patch test were positive, what allergens are you allergic to? What the biopsy revealed.<br>4. If you have not had patch tests performed or biopsy, do you notice deterioration of the skin condition after any factors, and if so, which ones?<br>5. Do you use dermatological treatment for the above-mentioned hand skin diseases, and if so, what kind?<br>6. What hand skin symptoms did you experience during exacerbations of the above-mentioned diseases?<br>7. Have you noticed any new hand skin symptoms while using disinfectants, and if so, which ones? |
| **Questions about identification of new symptoms or the severity of hand skin disease** | 1. How often during the day do you use hand disinfection?<br>2. What volume of hand disinfectant do you use during each application?<br>3. After washing your hands with soap and water, do you dry your hands thoroughly before applying hand disinfection?<br>4. Do you experience any pain or burning sensations during application of the hand disinfectant?<br>5. Do you experience any negative symptoms after applying the disinfectant, e.g. dryness, roughness, itching of the skin?<br>6. Since you started using hand disinfection in accordance to the SARS-CoV-2 pandemic, have you noticed any new hand skin symptoms that were never experienced before, and if so, which ones?<br>7. Have you noticed more frequent exacerbations of hand skin lesions since you started using hand disinfection in relation to the SARS-CoV-2 pandemic, and if so, which ones?<br>8. Since you started using hand disinfection during the SARS-CoV-2 pandemic, have you had to use |

medications to alleviate skin symptoms more frequently, if so, which ones?

9. Since the start of the use of hand disinfection in accordance to the SARS-CoV-2 pandemic, have you noticed that the period of use of symptom-relieving medication to achieve a state of remission of the disease has extended?
10. Since the start of the use of hand disinfection during the SARS-CoV-2 pandemic, have you had to seek more frequent medical advice/visits due to hand skin lesions?
11. Since you started using hand disinfection during the SARS-CoV-2 pandemic, have you had to change to advanced therapy to control skin symptoms?
12. Has the duration of remission (symptom-free period) during the COVID-19 pandemic significantly changed compared to the period before hand sanitizer use in accordance to the SARS-CoV-2 pandemic?
13. Before the period of use of hand disinfectants in relation to the SARS-CoV-2 pandemic, did you ever have microbial hand superinfections (bacterial, viral, fungal), and if so, which ones?
14. Before the SARS-CoV-2 pandemic period, had you been tested for bacterial and fungal hand skin superinfections, and if so, what was the result of the test?
15. Have you had microbial skin superinfections during the COVID-19 pandemic, and if so, which kind of superinfection?
16. Have you been tested for bacterial and fungal superinfections of hand skin during the SARS-CoV-2 pandemic, and if so, what was the result of the test?
17. Did you use moisturising products - emollients (e.g. creams, lotions, milks, ointments) - before the SARS-CoV-2 pandemic, and if so, how often?
18. Did you use moisturizers, emollients during the SARS-CoV-2 pandemic, and if so, how often?
19. Under what circumstances have you used moisturisers, emollients (e.g. before/after hand washing, before/after hand disinfection, when experienced dryness)?
20. 20. On a scale of 1 to 5, how would you rate the nuisance of using disinfection if 1 means disinfection had no negative effect on the quality of life, 5 – a significant impact of disinfection on the lowering quality of life?
